# Association of Fragmented Readmissions and Electronic Information Sharing With Discharge Destination Among Older Adults

**DOI:** 10.1001/jamanetworkopen.2023.13592

**Published:** 2023-05-16

**Authors:** Sara D. Turbow, Mohammed K. Ali, Steven D. Culler, Kimberly J. Rask, Molly M. Perkins, Carolyn K. Clevenger, Camille P. Vaughan

**Affiliations:** 1 Division of General Internal Medicine, Department of Medicine, Emory University School of Medicine, Atlanta, Georgia; 2 Department of Family and Preventive Medicine, Emory University School of Medicine, Atlanta, Georgia; 3 Hubert Department of Global Health, Rollins School of Public Health, Emory University, Atlanta, Georgia; 4 Department of Health Policy and Management, Rollins School of Public Health, Emory University, Atlanta, Georgia; 5 Alliant Health Solutions, Atlanta, Georgia; 6 Division of Geriatrics and Gerontology, Department of Medicine, Emory University School of Medicine, Atlanta, Georgia; 7 Nell Hodgson Woodruff School of Nursing, Emory University, Atlanta, Georgia; 8 Department of Veterans Affairs, Birmingham/Atlanta Geriatric Research Education and Clinical Center, Atlanta, Georgia

## Abstract

**Importance:**

When an older adult is hospitalized, where they are discharged is of utmost importance. Fragmented readmissions, defined as readmissions to a different hospital than a patient was previously discharged from, may increase the risk of a nonhome discharge for older adults. However, this risk may be mitigated via electronic information exchange between the admission and readmission hospitals.

**Objective:**

To determine the association of fragmented hospital readmissions and electronic information sharing with discharge destination among Medicare beneficiaries.

**Design, Setting, and Participants:**

This cohort study retrospectively examined data from Medicare beneficiaries hospitalized for acute myocardial infarction, congestive heart failure, chronic obstructive pulmonary disease, syncope, urinary tract infection, dehydration, or behavioral issues in 2018 and their 30-day readmission for any reason. The data analysis was completed between November 1, 2021, and October 31, 2022.

**Exposures:**

Same hospital vs fragmented readmissions and presence of the same health information exchange (HIE) at the admission and readmission hospitals vs no information shared between the admission and readmission hospitals.

**Main Outcomes and Measures:**

The main outcome was discharge destination following the readmission, including home, home with home health, skilled nursing facility (SNF), hospice, leaving against medical advice, or dying. Outcomes were examined for beneficiaries with and without Alzheimer disease using logistic regressions.

**Results:**

The cohort included 275 189 admission-readmission pairs, representing 268 768 unique patients (mean [SD] age, 78.9 [9.0] years; 54.1% female and 45.9% male; 12.2% Black, 82.1% White, and 5.7% other race and ethnicity). Of the 31.6% fragmented readmissions in the cohort, 14.3% occurred at hospitals that shared an HIE with the admission hospital. Beneficiaries with same hospital/nonfragmented readmissions tended to be older (mean [SD] age, 78.9 [9.0] vs 77.9 [8.8] for fragmented with same HIE and 78.3 [8.7] years for fragmented without HIE; *P* < .001). Fragmented readmissions were associated with 10% higher odds of discharge to an SNF (adjusted odds ratio [AOR], 1.10; 95% CI, 1.07-1.12) and 22% lower odds of discharge home with home health (AOR, 0.78; 95% CI, 0.76-0.80) compared with same hospital/nonfragmented readmissions. When the admission and readmission hospital shared an HIE, beneficiaries had 9% to 15% higher odds of discharge home with home health (patients without Alzheimer disease: AOR, 1.09 [95% CI, 1.04-1.16]; patients with Alzheimer disease: AOR, 1.15 [95% CI, 1.01-1.32]) compared with fragmented readmissions where information sharing was not available.

**Conclusions and Relevance:**

In this cohort study of Medicare beneficiaries with 30-day readmissions, whether a readmission is fragmented was associated with discharge destination. Among fragmented readmissions, shared HIE across admission and readmission hospitals was associated with higher odds of discharge home with home health. Efforts to study the utility of HIE for care coordination for older adults should be pursued.

## Key Points

**Question:**

What is the association between fragmented readmissions (ie, readmission to a different hospital than patient was discharged from) and health information exchange (HIE) and discharge destination?

**Findings:**

In this cohort study of 275 189 admission-readmission pairs of Medicare beneficiaries, fragmented readmissions were associated with a 10% higher odds of discharge to a skilled nursing facility and a 22% lower odds of discharge home with home health vs nonfragmented readmissions. In fragmented readmissions with shared HIE, beneficiaries had 9% to 15% higher odds of discharge home with home health vs fragmented readmissions in which information sharing was not available.

**Meaning:**

These findings indicate that fragmented readmissions may be associated with less favorable discharge destinations; however, HIE may mitigate information discontinuity present in fragmented readmissions.

## Teaser

This cohort study uses Medicare data to examine the association of fragmented hospital readmissions and information sharing with discharge destination in older adults.

## Introduction

Returning home after leaving the hospital is a major goal for most patients, particularly older adults. However, more than 40% of Medicare beneficiaries require postacute care following a hospitalization,^1^ a number that increased by 50% between 1996 and 2010^2^ and has continued to increase since.^3^ Up to 30% of patients discharged to postacute care require readmission to the hospital,^4^ which can further perpetuate the cycle of admissions, discharges to postacute care, and subsequent readmissions.^5,6,7,8^

Repeated hospitalizations, admissions to postacute care, and readmissions present opportunities for interhospital fragmentation of care, wherein a patient’s admission and readmission are to different hospitals. This type of care fragmentation is associated with higher in-hospital mortality,^9,10,11,12^ longer lengths of stay,^13,14^ and increased risk of subsequent readmissions.^9,15,16,17^ Because a patient’s medical history and record of previous hospitalization may not be available during a fragmented readmission, effective transitions of care may be challenging and may lead to higher rates of nonhome discharge than would be observed in a nonfragmented readmission.

Systems that can bridge the information discontinuity^18^ present in fragmented readmissions, namely shared electronic health records (EHRs) and health information exchanges (HIEs), may mitigate some of the negative effects of fragmented readmissions. Previous work has shown that HIEs may be associated with reduced costs,^19^ less duplication of procedures and imaging,^20^ and lower risk of subsequent readmissions^21,22,23^ and fragmented readmissions,^22^ among other outcomes.^24^

The association of information sharing with outcomes may differ across patient populations. If a patient can easily communicate their health history, the effect of outside sources of information on their clinical course may be lessened. However, if patients are not able to communicate their health history, such as is the case among some older adults with Alzheimer disease, electronic information sharing may have an outsize effect on outcomes. Previous work in the outpatient setting has shown that higher outpatient continuity of care resulted in fewer hospitalizations and emergency department visits among older adults with Alzheimer disease,^25^ a benefit that may extend to continuity of care with a hospital as well.

In this study, we analyzed the associations between fragmented readmissions and discharge destination among a sample of Medicare beneficiaries. We also assessed whether electronic health information sharing was associated with a patient’s odds of various discharge destinations, stratified by Alzheimer disease status.

## Methods

### Data Sources

This cohort study used the 2018 Medicare Provider Analysis and Review file, which was linked to the 2018 Medicare Master Beneficiary Summary and the Chronic Conditions Segment files to obtain additional beneficiary information. Hospital characteristics were obtained from the American Hospital Association (AHA) Annual Survey^26^ and the AHA Information Technology (IT) Supplement^27^ from 2017 to 2018. The study was approved by the institutional review board of the Emory University School of Medicine. with a waiver of informed consent due to no more than minimal risk to human participants. The study followed the Strengthening the Reporting of Observational Studies in Epidemiology (STROBE) reporting guideline.^28^

### Patients

Inpatient claims by Medicare beneficiaries who had a hospital admission for acute myocardial infarction, congestive heart failure, chronic obstructive pulmonary disease, pneumonia, dehydration, syncope, urinary tract infection, or behavioral issues in 2018 were obtained from the Centers for Medicare & Medicaid Services (see eAppendix in Supplement 1 for the *International Classification of Diseases, Tenth Revision* [*ICD-10*] codes used). These conditions were chosen because they are part of the Hospital Readmissions Reduction Program (acute myocardial infarction, congestive heart failure, chronic obstructive pulmonary disease, pneumonia)^29^ or are among the top 15 most common causes of hospitalization among older adults with Alzheimer disease (dehydration, syncope, urinary tract infection, behavioral issues).^30^ Although a beneficiary’s index admission was for 1 of the listed conditions, the readmission could be for any reason. We excluded beneficiaries who did not have a readmission in the data set and inpatient claims with missing beneficiary identification numbers or admissions resulting from hospital-to-hospital transfer.

From inpatient claims, we created admission-readmission pairs for each index admission and subsequent 30-day readmission. Multiple admission-readmission pairs could exist for a beneficiary if they had multiple 30-day readmissions. Beneficiaries who were listed as ever having a diagnosis of Alzheimer disease in the Chronic Conditions Segment^31^ were considered to have Alzheimer disease in this analysis.

### Primary Exposure: Fragmented Readmission

We first categorized all admission-readmission pairs as fragmented or as same hospital/nonfragmented. If the admission and readmission hospitals had different identification numbers in the Medicare data, the readmission was fragmented. If the identification numbers were the same, the readmission was determined to be to the same hospital/nonfragmented, which served as the comparison group for the primary analysis.

### Secondary Exposure: Type of Information Sharing

Among fragmented readmissions, we categorized information sharing based on the availability of electronic health information sharing at the admission and readmission hospitals based on the AHA IT Supplement.^27^ The AHA IT Supplement asks hospitals: “Please indicate your level of participation in a state, regional, and/or local [HIE] or health information organization [HIO].” Answer options were “do not know,” “HIE/HIO is not operational in my area,” “HIE/HIO is operational...but we are not participating,” or “HIE/HIO is operational...and we are participating.”^27^ If the hospital answered that HIE was operational in its area and it was participating in any AHA IT Supplement between 2017 and 2018, it was considered to have HIE capabilities for the purpose of this analysis. If a hospital had different or missing answers across the 2 years of data, we used the answer reflecting the hospital’s highest level of information sharing. We took this approach because each year of AHA IT Supplement data had more than 20% missingness in response to this question, but only 3.4% of hospitals that responded to the AHA IT Supplement survey had missing responses for both years. Because not all hospitals that responded to the AHA Annual Survey responded to the AHA IT Supplement survey, 17.1% of admission-readmission pairs in our analytic sample had missing HIE information (eTable 1 in Supplement 1). Justification for this approach is that it is unlikely for a hospital to move from participating in HIE to not participating in HIE during this time.

To determine which HIE a hospital participated in, we examined answers to the question: “Which of the following national health information networks does your hospital participate in?” Potential responses include “your EHR vendor’s network...” and “other,” which offered a free-text response option. We used these answers to categorize information sharing between different admission and readmission hospitals as participated in the same HIE, participated in different HIEs, 1 or both hospitals did not participate in an HIE, or 1 or both hospitals had missing HIE participation data.

In this analysis, we compared admission-readmission pairs in which both hospitals participated in the same HIE with admission-readmission pairs in which information sharing was not available because the admission and readmission hospitals had different HIEs, the admission and/or readmission hospital did not participate in HIE, or HIE data were missing; these 3 groups were combined to make the reference group. Because a beneficiary could have multiple admission-readmission pairs in this analysis, they could have a pair in more than 1 category of information sharing.

### Outcome: Discharge Destination Following the Readmission

The outcome of interest in this analysis was the discharge destination following the readmission. Potential discharge destinations were home, skilled nursing facility (SNF), home with home health, hospice, left against medical advice, died during the readmission, or other. Hospice could be either inpatient or home hospice. Other included discharge to law enforcement or psychiatric facilities.

### Covariates

Our analysis considered beneficiary demographic and clinical characteristics, as well as characteristics of the readmission hospital. Demographic characteristics included the beneficiary’s age, sex, and race and ethnicity (Black, White, or other). Race and ethnicity was identified in the Master Beneficiary Summary and uses Social Security Administration data,^31^ which is based on self-report. Other included Hispanic, Asian, North American Native, other, and unknown. Race and ethnicity were included as covariates in this analysis because patients from minoritized racial and ethnic groups may have disparities in where they are discharged to after hospitalization due to bias in the health care system. Baseline clinical characteristics included a frailty score, Charlson Comorbidity Index score, and the diagnosis-related group of the readmission. The frailty score ranged from 0 to 1, with higher scores indicating more frailty. It was calculated using a deficit accumulation model using 93 claims-based variables. The frailty score was originally developed and validated using *ICD-9* codes and has been updated with *ICD-10* codes.^32,33^

Readmission hospital characteristics included the number of hospital beds (<200, 200-399, ≥400), hospital ownership (government, church, nonprofit, for profit), hospital type (general medical/surgical, other), urban/rural status (metropolitan, micropolitan, rural), and whether the hospital was a teaching hospital. Urban/rural status was identified via the Rural Urban Commuting Area Code^34^ of the hospital. Hospitals were classified as teaching hospitals if they reported that they had programs accredited by the American Council of Graduate Medical Education, the American Osteopathic Association, or the Council of Teaching Hospitals or if they were affiliated with a medical school. Hospitals were categorized as either general medical/surgical or other, which included specialty hospitals.^26^

### Statistical Analysis

Descriptive statistics, including χ^2^ tests, *t* tests, and analysis of variance, were used to compare clinical and demographic characteristics between admission-readmission pairs across categories of information sharing; these were used to inform covariate selection for multivariable models. Covariates were checked for multicollinearity using the collin function in Stata (StataCorp LLC). Significance was set at a 1-sided *P* < .05.

To evaluate whether fragmented vs same hospital/nonfragmented readmissions were associated with discharge destination, we performed unadjusted and adjusted logistic regressions. Each discharge destination was evaluated using dummy variables. Regression analyses were adjusted for patient demographic characteristics, clinical characteristics, and readmission hospital characteristics. A fully adjusted model using all covariates was also created. Hospital referral region random effects were included in each model to account for geographic variation in SNFs and other discharge destination availability. Regression analyses were stratified a priori by Alzheimer disease status.

To examine the association between information sharing across the admission and readmission hospitals with discharge destination, we repeated unadjusted and adjusted logistic regressions comparing fragmented readmissions in which the admission and readmission hospitals participated in the same HIE with fragmented readmissions in which there was no information sharing available. As above, robust SEs clustered at the hospital level were used, and regression analyses were stratified a priori by Alzheimer disease status.

We also completed several sensitivity analyses. First, we included being admitted through the emergency department vs another route of admission as a covariate. Second, we removed admission-readmission pairs with missing HIE information from the composite reference group to account for potential misclassification bias introduced by these pairs. To estimate the associations of information sharing compared with nonfragmented readmissions, we compared same HIE and no information shared admission-readmission pairs with nonfragmented readmissions. Additionally, to test whether there was a differential association between information sharing and the type of hospice discharge, we examined home hospice and facility-based hospice as separate discharge destinations. All analyses were performed using SAS, version 9.4 (SAS Institute) and Stata, version 17 statistical software. The data analysis was completed between November 1, 2021, and October 31, 2022.

## Results

### Sample Development

**Figure 1.**
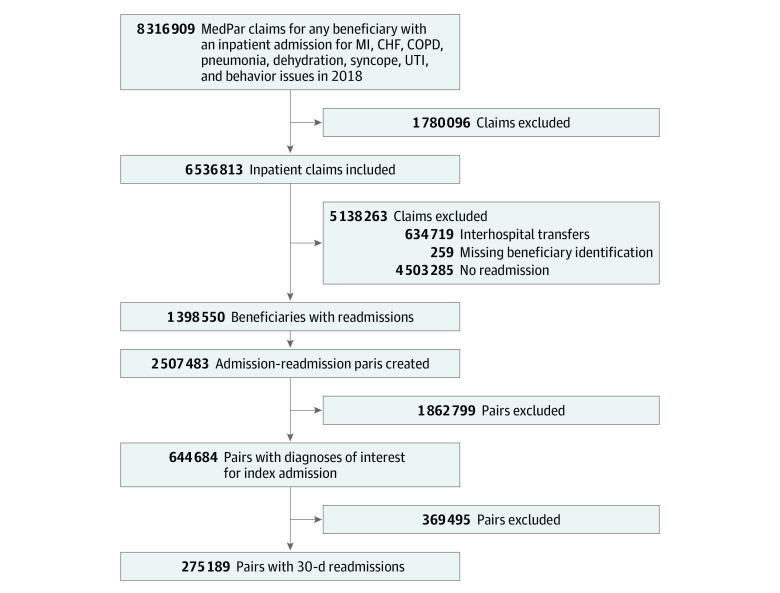
Study Flow Diagram CHF indicates congestive heart failure; COPD, chronic obstructive pulmonary disease; MedPar, Medicare Provider Analysis and Review; MI, myocardial infarction; UTI, urinary tract infection.

The 2018 Medicare sample had 8 316 909 claims (Figure 1). After we removed noninpatient claims, interhospital transfers, and observations missing beneficiary identification numbers, we created admission-readmission pairs (n = 2 507 483). After we limited the sample to 30-day readmissions and index admissions for the initial diagnoses of interest and removed pairs in which beneficiaries were listed as deceased after their index admission, 275 189 pairs (268 768 unique patients; mean [SD] age, 78.9 [9.0] years; 54.1% female and 45.9% male; 12.2% Black, 82.1% White, and 5.7% other race and ethnicity) remained in the final sample (Figure 1; Table).

**Table.** Demographic, Clinical, and Readmission Hospital Information of Admission-Readmission Pairs of Medicare Beneficiaries by Electronic Information Sharing Status, 2018^a^

	No. (%)				*P* value
	Total (N = 275 189)	Same hospital/nonfragmented readmissions (n = 188 106)	Fragmented readmissions/same HIE (n = 12 468)	Fragmented readmissions/no information sharing (n = 74 615)^b^	
Age, mean (SD) y	78.9 (9.0)	78.9 (9.0)	77.9 (8.8)	78.3 (8.7)	<.001
Sex					
Female	149 186 (54.2)	102 823 (54.7)	6444 (51.7)	39 919 (53.5)	<.001
Male	126 003 (45.8)	85 283 (45.3)	6024 (48.3)	34 696 (46.5)	
Race and ethnicity					
Black	33 663 (12.2)	21 706 (11.5)	1850 (14.8)	10 107 (13.5)	<.001
White	225 873 (82.1)	156 531 (83.2)	9976 (80.0)	59 366 (79.6)	
Other^c^	15 653 (5.7)	9869 (5.2)	642 (5.1)	5142 (6.7)	
Urban/rural status					
Metropolitan	236 344 (86.4)	162 113 (86.4)	11 817 (94.9)	62 414 (84.7)	<.001
Micropolitan	26 422 (9.7)	18 896 (10.1)	466 (3.7)	7060 (9.6)	
Rural	10 858 (3.9)	6526 (3.5)	172 (1.4)	4160 (5.6)	
Alzheimer disease	36 299 (13.2)	24 952 (13.3)	1587 (12.7)	9760 (13.1)	.13
Frailty score, mean (SD)	0.18 (0.03)	0.18 (0.03)	0.18 (0.03)	0.18 (0.03)	.16
CCI score, mean (SD)	3.9 (2.4)	4.0 (2.4)	3.9 (2.4)	3.8 (2.4)	<.001
Readmission via emergency department	212 996 (77.4)	150 673 (80.1)	8303 (66.6)	54 021 (72.4)	<.001
No. of beds in readmission hospital					
<200	141 532 (51.7)	92 735 (49.4)	5510 (44.2)	43 287 (58.8)	<.001
200-399	67 630 (24.7)	48 318 (25.8)	3027 (24.3)	16 285 (22.1)	
≥400	64 462 (23.6)	46 482 (24.8)	3918 (31.5)	14 062 (19.1)	
Readmission hospital ownership					
Government	28 692 (10.5)	20 515 (10.9)	954 (7.7)	7223 (9.8)	<.001
Church	34 066 (12.5)	23 143 (12.3)	1658 (13.3)	9265 (12.7)	
Nonprofit	165 034 (60.3)	119 324 (63.7)	8347 (67.0)	37 363 (50.8)	
For profit	45 686 (16.7)	24 455 (13.0)	1496 (12.0)	19 735 (26.8)	
Readmission hospital type					
General medical/surgical	269 740 (98.9)	186 948 (99.8)	11 987 (96.6)	70 805 (96.9)	<.001
Other	3074 (1.1)	401 (0.2)	427 (3.4)	2246 (3.1)	
Readmission hospital teaching status					
Yes	193 953 (70.9)	135 049 (72.0)	9874 (79.3)	49 030 (66.6)	<.001
Readmission hospital region					
Midwest	65 108 (23.9)	48 981 (26.2)	3209 (25.8)	12 918 (17.6)	<.001
North	54 583 (20.0)	37 362 (20.0)	2810 (22.6)	14 411 (19.7)	
South	115 657 (42.4)	78 546 (42.0)	5371 (43.1)	31 740 (43.3)	
West	37 488 (13.7)	22 276 (11.9)	1063 (8.5)	14 149 (19.3)	
Discharge destination from readmission					
Home	78 309 (28.5)	52 830 (28.1)	3253 (28.3)	21 956 (29.4)	<.001
SNF	82 286 (29.9)	56 249 (29.9)	3804 (30.5)	22 233 (29.8)	.27
Home with home health	64 778 (23.5)	46 276 (24.6)	2868 (23.0)	15 634 (20.9)	<.001
Hospice	17 442 (6.3)	12 415 (6.6)	753 (6.0)	4274 (5.7)	<.001
Left against medical advice	1756 (0.6)	992 (0.5)	93 (0.7)	671 (0.9)	<.001
Died	15 050 (5.5)	9898 (5.3)	701 (5.6)	4451 (6.0)	<.001
Other	15 568 (5.7)	9446 (5.0)	726 (5.8)	5396 (7.2)	<.001

Abbreviations: CCI, Charlson Comorbidity Index; HIE, health information exchange; SNF, skilled nursing facility.

^a^ Comparisons are across categories of information sharing (same hospital/nonfragmented readmissions vs fragmented/same HIE vs fragmented/no information sharing).

^b^ Includes admission and readmission hospital with different HIEs, 1 or both with no HIE, 1 or both with missing HIE.

^c^ Other race and ethnicity included Asian, Hispanic, North American Native, other, and unknown.

### Hospital and Patient Characteristics

Overall, 31.6% of pairs had fragmented readmissions. Compared with beneficiaries with fragmented readmissions that shared an HIE and those that did not, beneficiaries with same hospital/nonfragmented readmissions tended to be older (mean [SD] age, 78.9 [9.0] vs 77.9 [8.8] and 78.3 [8.7] years; *P* < .001) and were more likely to be White (83.2% vs 80.0% and 79.6%; *P* < .001) vs Black (11.5% vs 14.8% and 13.5%) and other race and ethnicity (5.2% vs 5.1% and 6.7%). Distribution by sex between same hospital/nonfragmented and fragmented readmissions that shared and did not share an HIE was 54.7%, 51.7, and 53.5% female patients and 45.3%, 48.3%, and 46.5% male patients, respectively. Beneficiaries with fragmented readmissions to hospitals that shared an HIE were more likely to be in a metropolitan area (94.9% vs 84.7%; *P* < .001) and to have a readmission to a teaching hospital (79.3% vs 66.6%; *P* < .001) than beneficiaries with fragmented readmissions to hospitals that did not share an HIE (Table). Beneficiary demographic and clinical characteristics as well as readmission hospital characteristics by subcategories of hospitals that did not share an HIE (ie, different HIEs, no HIE, HIE information missing) are shown in eTable 1 in Supplement 1.

### Fragmentation of Care and Discharge Destination

**Figure 2.**
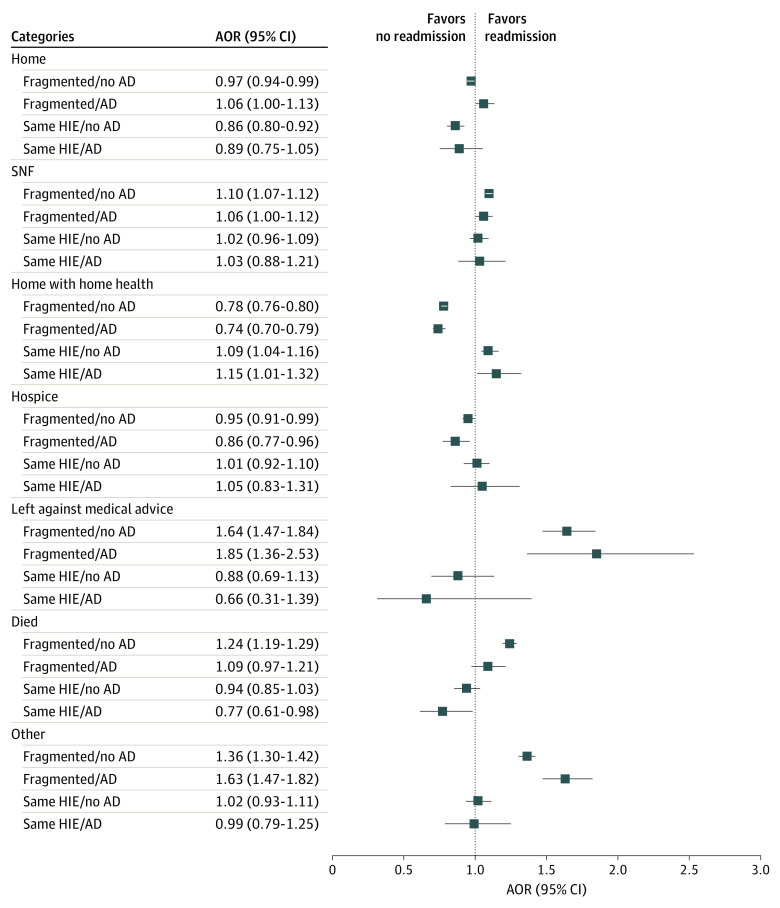
Associations Among Fragmented Readmissions, Electronic Information Sharing, and Discharge Destinations, Stratified by Alzheimer Disease (AD) Diagnosis, 2018 Medicare Beneficiaries The reference group for fragmented models was same hospital/nonfragmented readmissions. The reference group for health information exchange (HIE) models was fragmented admission-readmission pairs where the hospitals did not participate in the same HIE. AOR indicates adjusted odds ratio; SNF, skilled nursing facility.

Compared with beneficiaries with a same hospital/nonfragmented readmission, adjusting for demographic, clinical, and readmission hospital characteristics, older adults without Alzheimer disease who experienced a fragmented readmission had 10% higher odds of discharge to an SNF (adjusted odds ratio [AOR], 1.10; 95% CI, 1.07-1.12), 64% higher odds of leaving against medical advice (AOR, 1.64; 95% CI, 1.47-1.84), and 24% higher odds of dying (AOR, 1.24; 95% CI, 1.19-1.29). They also had lower odds of being discharged home (AOR, 0.97; 95% CI, 0.94-0.99), home with home health (AOR, 0.78; 95% CI, 0.76-0.80), or to hospice (AOR, 0.95; 95% CI, 0.91-0.99) (Figure 2; eTable 2 in Supplement 1).

Beneficiaries with Alzheimer disease had higher odds of discharge home (AOR, 1.06; 95% CI, 1.00-1.13), to an SNF (AOR, 1.06; 95% CI, 1.00-1.12), and of leaving against medical advice (AOR, 1.85; 95% CI, 1.36-2.53) when their readmission was fragmented vs same hospital/nonfragmented in fully adjusted models (Figure 2; eTable 3 in Supplement 1). Beneficiaries with Alzheimer disease experiencing fragmented readmissions also had lower odds of discharge home with home health (AOR, 0.74; 95% CI, 0.70-0.79) or to hospice (AOR, 0.86; 95% CI, 0.77-0.96); there was no difference observed in the odds of dying during the readmission (AOR, 1.09; 95% CI, 0.97-1.21).

### Information Sharing and Discharge Destination

Among beneficiaries with fragmented readmissions, 14.3% were readmitted to a different hospital that shared an HIE with the admission hospital. In fully adjusted models, beneficiaries without Alzheimer disease and fragmented readmissions in which the admission and readmission hospital shared the same HIE had 14% lower odds of being discharged home (AOR, 0.86; 95% CI, 0.80-0.92) but 9% higher odds of being discharged home with home health (AOR, 1.09; 95% CI, 1.04-1.16) compared with beneficiaries with fragmented readmissions where the admission and readmission hospitals did not share an HIE (Figure 2; eTable 4 in Supplement 1). Beneficiaries with Alzheimer disease similarly had 15% higher odds of discharge home with home health when readmitted to a different hospital that shared an HIE with the index hospital (AOR, 1.15; 95% CI, 1.01-1.32) compared with hospitals that did not share HIEs. Additionally, beneficiaries with AD had 23% lower odds of dying during the readmission if the admission and readmission hospitals shared an HIE (AOR, 0.77; 95% CI, 0.61-0.98) compared with fragmented readmissions where the hospitals did not share an HIE (Figure 2; eTable 5 in Supplement 1).

### Sensitivity Analysis

When we included emergency department origin as a covariate, removed fragmented admission-readmission pairs that had missing HIE data, and compared fragmented admission-readmission pairs that shared an HIE and those that did not with same hospital/nonfragmented admission-readmission pairs, the trends were similar to those seen in the main analysis (eTables 6-10 in Supplement 1). When hospice discharge was separated into home and facility-based hospice, beneficiaries without Alzheimer disease with fragmented readmissions where an HIE was shared had 15% lower odds of discharge to home hospice (AOR, 0.85; 95% CI, 0.74-0.97) but 15% higher odds of discharge to a facility-based hospice (AOR, 1.15; 95% CI, 1.02-1.30) after adjusting for demographic, clinical, and hospital characteristics (eTable 11 in Supplement 1).

## Discussion

In this cohort study, fragmented readmissions were common (>30% of readmissions), and the association between these fragmented readmissions and discharge destination was notable. For older adults with and without Alzheimer disease, compared with readmission at the same hospital, a fragmented readmission was associated with higher odds of discharge to an SNF by 6% to 10%, lower odds of discharge home with home health and to hospice by 22% to 26% and 5% to 14%, respectively, and higher odds of leaving against medical advice between 64% and 85%.

There are patient-, hospital-, and system-level reasons why a patient may have a fragmented readmission. At the patient level, fragmented readmissions may be influenced by a patient’s location or geography,^35,36,37^ their family’s wishes, acuity of their illness, and dissatisfaction with previous care.^38^ A patient’s diagnosis and illness severity, the quality of care, need for specialist care, and insurance payer can affect whether they return to their previous hospital. Previous work has shown that at the hospital and health care system level, density of hospitals and ambulance use are also associated with fragmented readmissions.^39,40^

Notably, more than 85% of patients with a fragmented readmission were readmitted to hospitals that did not share an HIE with the admission hospital. While information sharing via HIE in fragmented readmissions was associated with discharge destination, it was not enough to completely mitigate the association between fragmentation and discharge destination in most cases.

One exception to the trend was home health; beneficiaries with and without Alzheimer disease who had fragmented readmissions in which both the admission and readmission hospitals shared an HIE had 9% to 15% increased odds of going home with home health compared with fragmented readmissions in which the admission and readmission hospitals did not share an HIE. This association may be due to the electronically available health information; for example, accessing notes from a previous hospitalization or from outpatient visits may provide information about a patient’s goals of care^41,42^ (ie, to avoid discharge to an SNF) or their baseline functional status.^43^

Fragmented readmissions were associated with 24% higher odds of dying during the readmission in beneficiaries without Alzheimer disease, but there was no statistically significant difference in the odds of dying during the readmission in beneficiaries with Alzheimer disease. When those fragmented readmissions occurred at hospitals that shared an HIE, beneficiaries with Alzheimer disease had a 23% lower odds of dying during the readmission compared with fragmented readmissions to hospitals that did not share an HIE (AOR, 0.77; 95% CI, 0.61-0.98). Older adults with Alzheimer disease may be particularly sensitive to poor care coordination, so the observation of a positive association with in-hospital mortality when information exchange is present is encouraging. While our data set does not define HIE pairs beyond hospitals, 1 potential reason for this observation may be that information exchange is not limited to hospitals; HIEs and other electronic information exchange systems also exist between the inpatient and outpatient settings and between hospitals and long-term-care facilities, which could further facilitate care coordination.

These results have implications beyond an individual patient and discharge destination. Beyond the paramount importance of being able to honor a patient’s wishes, different discharge destinations add different stressors to patients and the health care system. Discharges to SNFs, for example, are associated with higher costs^3,44^ and an increased risk of subsequent readmission.^45,46^ Discharges to home with home health support are increasing,^47^ and patients and payers may prefer these to inpatient postacute care facilities. To provide each patient with the best possible care, including preventing unnecessarily intense care, clinicians should be encouraged to consider the effect of fragmentation and to seek additional information about care delivered elsewhere.

Hospitals that have HIE functionality may be different than those that do not. Previous work has found that hospitals that are part of health care systems are more likely to participate in electronic information exchange,^48,49^ as are hospitals in areas with a dominant EHR vendor,^50^ larger hospitals, and nonprofit hospitals.^51^ Some of these characteristics may be associated with quality of care delivered,^52^ the rate of fragmented readmissions, and discharge destination.

### Limitations

This study has several limitations. A key limitation of the analysis is that hospital-level availability of HIE may not reflect clinician use of HIE. This limitation is common in observational studies using secondary data on HIE topics.^22,23,53,54^ In previous studies, a lack of clinically useful information^55,56^ was 1 reason that providers reported accessing HIEs in only 10% to 20% of clinical encounters.^19,57^ However, what may be clinically useful in the care of a patient at the beginning of an acute care episode may be different from what is useful for discharge planning. Specific investigations into how HIEs are used in care coordination vs clinical decision making could shed more light on this issue. As HIE use continues to expand, in part due to federal priorities,^58^ it will be increasingly important to understand how and when clinicians use HIE and what effect the information contained in the HIE has on clinical decision making, care coordination, and patient outcomes.

Another limitation is how hospitals responded to the AHA IT Supplement survey. Despite including 2 years of data, several hospitals had missing answers to the questions around HIE availability at the hospital level or did not respond to the survey at all (eTable 2 in Supplement 1). While this missingness of data could present a source of misclassification bias, our sensitivity analyses where missing or incomplete data were removed showed overall similar results to the primary analysis. Additionally, by using cross-sectional survey data, we were not able to measure the effect of changes to a hospital’s HIE participation that may have occurred partway through the year.

## Conclusions

The data from this cohort study show an association between fragmented care and suboptimal discharge destinations, which may be partly due to information discontinuity that may impede care coordination. Our analysis shows that electronic information exchange may be a potential solution and that the information contained in HIEs may bridge the gap between different hospitals, facilitating more effective transitions of care to mitigate the effects of fragmented care on discharge destinations.

## Notes

Supplement 1. **eAppendix.** *International Classification of Diseases, Tenth Revision* (*ICD-10*) and Diagnosis-Related Group (DRG) Codes Used for Index Admissions **eTable 1.** Demographic, Clinical, and Readmission Hospital Information of Admission-Readmission Pairs of Medicare Beneficiaries by Electronic Information Sharing Status, by No Information Share Subcategories, 2018 **eTable 2.** Association of Fragmentation Status and Discharge Destination From Readmission, Medicare Beneficiaries Without Alzheimer Disease, 2018 **eTable 3.** Association of Fragmentation Status and Discharge Destination From Readmission, Medicare Beneficiaries With Alzheimer Disease, 2018 **eTable 4.** Association of Health Information Exchange Status and Discharge Destination From Readmission, Medicare Beneficiaries Without Alzheimer Disease, 2018 **eTable 5.** Association of Health Information Exchange Status and Discharge Destination From Readmission, Medicare Beneficiaries With Alzheimer Disease, 2018 **eTable 6.** Association of Health Information Exchange Status and Discharge Destination From Readmission, Medicare Beneficiaries Without Alzheimer Disease, 2018 **eTable 7.** Association of Health Information Exchange Status and Discharge Destination From Readmission, Medicare Beneficiaries With Alzheimer Disease, 2018 **eTable 8.** Association of Health Information Exchange (HIE) Status and Discharge Destination From Readmission, Missing HIE Information Pairs Removed, Medicare Beneficiaries With and Without Alzheimer Disease, 2018 **eTable 9.** Association of Health Information Exchange Status and Discharge Destination From Readmission Compared With Same Hospital/Nonfragmented Readmissions, Medicare Beneficiaries Without Alzheimer Disease, 2018 **eTable 10.** Association of Health Information Exchange Status and Discharge Destination From Readmission Compared With Same Hospital/Nonfragmented Readmissions, Medicare Beneficiaries With Alzheimer Disease, 2018 **eTable 11.** Association of Fragmented Readmissions and Discharge to Home Hospice or Facility-Based Hospice From Readmission, Medicare Beneficiaries, 2018

Supplement 2.
Data Sharing Statement
