# Supplementary material for: Association of Fragmented Readmissions and Electronic Information Sharing With Discharge Destination Among Older Adults
Source: JAMA Netw Open. 2023 May 16;6(5):e2313592. doi: 10.1001/jamanetworkopen.2023.13592 (PMC10189568; doi:10.1001/jamanetworkopen.2023.13592)
Supplement: Supplement 1. — eAppendix. International Classification of Diseases, Tenth Revision (ICD-10) and Diagnosis-Related Group (DRG) Codes Used for Index Admissions eTable 1. Demographic, Clinical, and Readmission Hospital Information of Admission-Readmission Pairs of Medicare Beneficiaries by Electronic Information Sharing Status, by No Information Share Subcategories, 2018 eTable 2. Association of Fragmentation Status and Discharge Destination From Readmission, Medicare Beneficiaries Without Alzheimer Disease, 2018 eTable 3. Association of Fragmentation Status and Discharge Destination From Readmission, Medicare Beneficiaries With Alzheimer Disease, 2018 eTable 4. Association of Health Information Exchange Status and Discharge Destination From Readmission, Medicare Beneficiaries Without Alzheimer Disease, 2018 eTable 5. Association of Health Information Exchange Status and Discharge Destination From Readmission, Medicare Beneficiaries With Alzheimer Disease, 2018 eTable 6. Association of Health Information Exchange Status and Discharge Destination From Readmission, Medicare Beneficiaries Without Alzheimer Disease, 2018 eTable 7. Association of Health Information Exchange Status and Discharge Destination From Readmission, Medicare Beneficiaries With Alzheimer Disease, 2018 eTable 8. Association of Health Information Exchange (HIE) Status and Discharge Destination From Readmission, Missing HIE Information Pairs Removed, Medicare Beneficiaries With and Without Alzheimer Disease, 2018 eTable 9. Association of Health Information Exchange Status and Discharge Destination From Readmission Compared With Same Hospital/Nonfragmented Readmissions, Medicare Beneficiaries Without Alzheimer Disease, 2018 eTable 10. Association of Health Information Exchange Status and Discharge Destination From Readmission Compared With Same Hospital/Nonfragmented Readmissions, Medicare Beneficiaries With Alzheimer Disease, 2018 eTable 11. Association of Fragmented Readmissions and Discharge to Home Hospice or Facil [file jamanetwopen-e2313592-s001.pdf]

Supplemental Online Content

Turbow SD, Ali MK, Culler SD, et al. Association of fragmented readmissions and electronic information sharing with discharge destination among older adults. *JAMA Netw Open*. 2023;6(5):e2313592. doi:10.1001/jamanetworkopen.2023.13592

eAppendix. *International Classification of Diseases, Tenth Revision* (*ICD-10*) and Diagnosis-Related Group (DRG) Codes Used for Index Admissions

eTable 1. Demographic, Clinical, and Readmission Hospital Information of Admission-Readmission Pairs of Medicare Beneficiaries by Electronic Information Sharing Status, by No Information Share Subcategories, 2018

eTable 2. Association of Fragmentation Status and Discharge Destination From Readmission, Medicare Beneficiaries Without Alzheimer Disease, 2018

eTable 3. Association of Fragmentation Status and Discharge Destination From Readmission, Medicare Beneficiaries With Alzheimer Disease, 2018

eTable 4. Association of Health Information Exchange Status and Discharge Destination From Readmission, Medicare Beneficiaries Without Alzheimer Disease, 2018

eTable 5. Association of Health Information Exchange Status and Discharge Destination From Readmission, Medicare Beneficiaries With Alzheimer Disease, 2018

eTable 6. Association of Health Information Exchange Status and Discharge Destination From Readmission, Medicare Beneficiaries Without Alzheimer Disease, 2018

eTable 7. Association of Health Information Exchange Status and Discharge Destination From Readmission, Medicare Beneficiaries With Alzheimer Disease, 2018

eTable 8. Association of Health Information Exchange (HIE) Status and Discharge Destination From Readmission, Missing HIE Information Pairs Removed, Medicare Beneficiaries With and Without Alzheimer Disease, 2018

eTable 9. Association of Health Information Exchange Status and Discharge Destination From Readmission Compared With Same Hospital/Nonfragmented Readmissions, Medicare Beneficiaries Without Alzheimer Disease, 2018

eTable 10. Association of Health Information Exchange Status and Discharge Destination From Readmission Compared With Same Hospital/Nonfragmented Readmissions, Medicare Beneficiaries With Alzheimer Disease, 2018

eTable 11. Association of Fragmented Readmissions and Discharge to Home Hospice or Facility-Based Hospice From Readmission, Medicare Beneficiaries, 2018

This supplemental material has been provided by the authors to give readers additional information about their work.

© 2023 Turbow SD et al. *JAMA Network Open.*

eAppendix. ***International Classification of Diseases*****,** ***Tenth Revision*** **(*****ICD-10*****) and Diagnosis-Related Group (DRG) Codes Used for Index Admissions**

ICD-10 Codes

I2101
I2102
I2109
I2111
I2119
I2121
I2129
I213
I214
I220
I221
I222
I228
I229
I0981
I110
I130
I132
I501
I5020
I5021
I5022
I5023
I5030
I5031
I5032

© 2023 Turbow SD et al. *JAMA Network Open.*

I5033
I5040
I5041
I5042
I5043
I509
R570
R579
J411
J418
J42
J430
J431
J432
J438
J439
J440
J441
J449
J470
J471
J479
J684
J688
J689
Q334
B330
J09X1
J09X2

© 2023 Turbow SD et al. *JAMA Network Open.*

J1000
J1001
J1008
J101
J1100
J1108
J120
J121
J122
J123
J1281
J1289
J129
J13
J14
J153
J154
J157
J159
J160
J168
J180
J181
J188
J189
J920
J929
J941
J949

© 2023 Turbow SD et al. *JAMA Network Open.*

R091
E860
E861
E869
E870
I951
I952
I953
I9581
R55
A1810
A1811
A1812
A1813
A3685
A5275
A5401
A5611
A5619
A985
B650
B901
N10
N110
N118
N119
N12
N135
N136

© 2023 Turbow SD et al. *JAMA Network Open.*

N151
N2884
N2885
N2886
N3000
N3001
N3010
N3011
N3020
N3021
N3030
N3031
N3080
N3081
N3090
N3091
N340
N342
N343
N390
F05
R442
R443

DRGs

190
191
192
193
194

© 2023 Turbow SD et al. *JAMA Network Open.*

195
280
281
282
283
284
285
291
292
293
312
640
641
689
690
880
881

© 2023 Turbow SD et al. *JAMA Network Open.*

eTable 1. Demographic, Clinical, and Readmission Hospital Information of Admission-Readmission Pairs of Medicare Beneficiaries by Electronic Information Sharing Status, by No Information Share Subcategories, 2018

		Total (n=275,189)	Same Hospital/ Nonfragmented Readmissions (N=188,106)	Same HIE* (N=12,468)	Different HIEs (N=16,706)	No HIE (N=10,709)	HIE information missing (N=47,200)	p
Age (mean)		78.9 ± 9.0	78.9 ± 9.0	77.9 ± 8.8	77.9 ± 8.8	78.0 ± 8.7	78.4 ± 8.9	<0.001
Sex	Female	54.1%	54.7%	51.7%	52.8%	52.2%	54.0%	<0.001
	Male	45.9%	45.3%	48.3%	47.2%	47.8%	46.0%	
Race	Black	12.2%	11.5%	14.8%	16.1%	14.6%	12.4%	<0.001
	Other**	5.7%	5.2%	5.1%	6.2%	5.9%	7.3%	
	White	82.1%	83.2%	80.0%	77.7%	79.4%	80.3%	
Urban/rural Status	Metro	86.4%	86.4%	94.9%	92.3%	87.5%	81.4%	<0.001
	Micro	9.7%	10.1%	3.7%	5.4%	8.9%	11.2%	
	Rural	3.9%	3.5%	1.4%	2.3%	3.6%	7.3%	
Alzheimer's Disease		13.2%	13.3%	12.7%	13.2%	12.9%	13.1%	0.32
Frailty Score		0.18 ± 0.03	0.18 ± 0.03	0.18 ± 0.03	0.18 ± 0.03	0.18 ± 0.03	0.18 ± 0.03	0.16
Charlson Comorbidity Index		3.9 ± 2.4	4.0 ± 2.4	3.9 ± 2.4	3.8 ± 2.4	3.8 ± 2.4	3.8 ± 2.4	<0.001
ICU stay Readmission		35.3%	33.8%	37.3%	36.7%	37.7%	39.7%	<0.001
Bedsize of Readmission hospital	<200 beds	51.7%	49.4%	44.2%	44.4%	50.6%	65.8%	<0.001
	200-399 beds	24.7%	25.8%	24.3%	25.9%	23.6%	20.4%	
	≥400 beds	23.6%	24.8%	31.5%	29.7%	25.8%	13.7%	
Readmission Hospital Ownership	Government	10.5%	10.9%	7.7%	9.5%	13.5%	9.1%	<0.001
	Church	12.5%	12.3%	13.3%	12.7%	9.4%	13.3%	
	Nonprofit	63.7%	67.0%	60.7%	57.5%	45.7%	45.7%	
	For-profit	16.7%	13.0%	12.0%	17.1%	19.7%	31.9%	
Readmission Hospital Type	General Medical/ Surgical	98.9%	99.8%	96.6%	96.4%	95.2%	97.5%	<0.001

© 2023 Turbow SD et al. *JAMA Network Open.*

		Total (n=275,189)	Same Hospital/ Nonfragmented Readmissions (N=188,106)	Same HIE* (N=12,468)	Different HIEs (N=16,706)	No HIE (N=10,709)	HIE information missing (N=47,200)	p
	Other	1.1%	0.2%	3.4%	3.6%	4.8%	2.5%	
Readmission Hospital Teaching Status	Yes	70.9%	72.0%	79.3%	77.4%	69.2%	62.1%	<0.001
Discharge Destination from readmission	Home	28.5%	28.1%	28.3%	28.7%	31.0%	29.3%	<0.001
	Skilled Nursing Facility	29.9%	29.9%	30.5%	30.5%	29.4%	29.6%	0.08
	Home with Home Health	23.5%	24.6%	23.0%	21.7%	19.2%	21.1%	<0.001
	Hospice	6.3%	6.6%	6.0%	5.3%	5.9%	5.8%	<0.001
	Against Medical Advice	0.6%	0.5%	0.7%	1.0%	1.1%	0.8%	<0.001
	Died	5.5%	5.3%	5.6%	6.3%	6.1%	5.8%	<0.001
	Other	5.7%	5.0%	5.8%	6.5%	7.3%	7.5%	<0.001

*HIE = health information exchange

**“Other” race includes: Hispanic, Asian, North American Native, Unknown, Other

© 2023 Turbow SD et al. *JAMA Network Open.*

Regression Models for Main Analysis

eTable 2. Association of Fragmentation Status and Discharge Destination From Readmission, Medicare Beneficiaries Without Alzheimer Disease, 2018

Discharge Destination	Unadjusted	Demographics & Baseline Clinical	Hospital	Full
Home	1.05 (1.03, 1.07)	0.98 (0.96, 1.00)	1.04 (1.01, 1.06)	0.97 (0.94, 0.99)
Skilled Nursing Facility	1.03 (1.01, 1.05)	1.07 (1.05, 1.10)	1.06 (1.03, 1.08)	1.10 (1.07, 1.12)
Home w/ Home Health	0.81 (0.79, 0.83)	0.81 (0.79, 0.83)	0.78 (0.76, 0.80)	0.78 (0.76, 0.80)
Hospice	0.85 (0.82, 0.89)	0.92 (0.88, 0.96)	0.87 (0.84, 0.91)	0.95 (0.91, 0.99)
Against Medical Advice	1.76 (1.58, 1.96)	1.62(1.45, 1.81)	1.79 (1.61, 1.99)	1.64 (1.47, 1.84)
Died	1.17 (1.12, 1.22)	1.22 (1.17, 1.28)	1.18 (1.13, 1.23)	1.24 (1.19, 1.29)
Other	1.31 (1.26, 1.37)	1.30 (1.24, 1.35)	1.38 (1.32, 1.44)	1.36 (1.30, 1.42)

Reference: nonfragmented/same hospital readmissions; each model includes hospital referral region random effects
Unadjusted
Model 1: demographics (age, sex, race), clinical (frailty score, Charlson comorbidity index, reason for readmission [DRG of readmission])
Model 2: hospital (urban/rural, size, ownership, control, teaching; each for readmission hospital)
Model 3: Full model—all above covariates included

eTable 3. Association of Fragmentation Status and Discharge Destination From Readmission, Medicare Beneficiaries With Alzheimer Disease, 2018

Discharge Destination	Unadjusted	Demographics & Baseline Clinical	Hospital	Full
Home	1.12 (1.06, 1.19)	1.05 (0.99, 1.12)	1.13 (1.07, 1.20)	1.06 (1.00, 1.13)
Skilled Nursing Facility	1.02 (0.96, 1.08)	1.05 (1.00, 1.11)	1.03 (0.97, 1.09)	1.06 (1.00, 1.12)
Home w/ Home Health	0.78 (0.73, 0.83)	0.78 (0.73, 0.83)	0.74 (0.70, 0.79)	0.74 (0.70, 0.79)
Hospice	0.79 (0.71, 0.87)	0.84 (0.76, 0.94)	0.80 (0.72, 0.89)	0.86 (0.77, 0.96)
Against Medical Advice	2.03 (1.51, 2.74)	1.80 (1.33, 2.44)	2.10 (1.55, 2.84)	1.85 (1.36, 2.53)
Died	1.02 (0.92, 1.13)	1.06 (0.95, 1.18)	1.03 (0.93, 1.15)	1.09 (0.97, 1.21)
Other	1.55 (1.40, 1.72)	1.52 (1.37, 1.68)	1.67 (1.50, 1.86)	1.63 (1.47, 1.82)

Reference: nonfragmented/same hospital readmissions; each model includes hospital referral region random effects Unadjusted
Model 1: demographics (age, sex, race), clinical (frailty score, Charlson comorbidity index, reason for readmission [DRG of readmission])
Model 2: hospital (urban/rural, size, ownership, control, teaching; each for readmission hospital)
Model 3: Full model—all above covariates included

© 2023 Turbow SD et al. *JAMA Network Open.*

eTable 4. Association of Health Information Exchange Status and Discharge Destination From Readmission, Medicare Beneficiaries Without Alzheimer Disease, 2018

Discharge Destination	Unadjusted	Demographics & Baseline Clinical	Hospital	Full
Home	0.97 (0.92, 1.01)	0.94 (0.80, 0.99)	0.94 (0.90, 0.99)	0.86 (0.80, 0.92)
Skilled Nursing Facility	0.99 (0.94, 1.04)	1.01 (0.96, 1.06)	1.00 (0.95, 1.04)	1.02 (0.96, 1.09)
Home w/ Home Health	1.13 (1.08, 1.19)	1.13 (1.07, 1.19)	1.10 (1.04, 1.16)	1.09 (1.04, 1.16)
Hospice	1.02 (0.93, 1.12)	1.04 (0.95, 1.14)	1.00 (0.91, 1.09)	1.01 (0.92, 1.10)
Against Medical Advice	0.93 (0.73, 1.18)	0.89 (0.70, 1.14)	0.90 (0.71, 1.15)	0.88 (0.69, 1.13)
Died	0.97 (0.89, 1.06)	0.97 (0.89, 1.07)	0.93 (0.85, 1.02)	0.94 (0.85, 1.03)
Other	0.83 (0.76, 0.91)	0.83 (0.76, 0.91)	1.02 (0.93, 1.12)	1.02 (0.93, 1.11)

Reference: fragmented admission-readmission pairs where the admission and readmission hospitals do not participate in the same HIE; each model includes hospital referral region random effects

Unadjusted

Model 1: demographics (age, sex, race), clinical (frailty score, Charlson comorbidity index, reason for readmission [DRG of readmission])

Model 2: hospital (urban/rural, size, ownership, control, teaching; each for readmission hospital)

Model 3: Full model—all above covariates included

© 2023 Turbow SD et al. *JAMA Network Open.*

eTable 5. Association of Health Information Exchange Status and Discharge Destination from Readmission, Medicare Beneficiaries With Alzheimer Disease, 2018

Discharge Destination	Unadjusted	Demographics & Baseline Clinical	Hospital	Full
Home	0.97 (0.86, 1.10)	0.97 (0.85, 1.10)	0.93 (0.82, 1.06)	0.89 (0.75, 1.05)
Skilled Nursing Facility	0.99 (0.88, 1.12)	1.01 (0.90, 1.14)	0.99 (0.88, 1.11)	1.03 (0.88, 1.21)
Home w/ Home Health	1.18 (1.03, 1.34)	1.18 (1.02, 1.33)	1.16 (1.02, 1.33)	1.15 (1.01, 1.32)
Hospice	1.09 (0.87, 1.35)	1.05 (0.85, 1.31)	1.09 (0.87, 1.36)	1.05 (0.83, 1.31)
Against Medical Advice	0.73 (0.36, 1.48)	0.66 (0.31, 1.38)	0.72 (0.36, 1.47)	0.66 (0.31, 1.39)
Died	0.84 (0.67, 1.06)	0.81 (0.64, 1.02)	0.81 (0.64, 1.03)	0.77 (0.61, 0.98)
Other	0.80 (0.63, 1.00) (p=0.050)	0.81 (0.65, 1.02)	0.98 (0.78, 1.23)	0.99 (0.79, 1.25)

Reference: fragmented admission-readmission pairs where the admission and readmission hospitals do not participate in the same HIE; each model includes hospital referral region random effects

Unadjusted

Model 1: demographics (age, sex, race), clinical (frailty score, Charlson comorbidity index, reason for readmission [DRG of readmission])

Model 2: hospital (urban/rural, size, ownership, control, teaching; each for readmission hospital)

Model 3: Full model—all above covariates included

© 2023 Turbow SD et al. *JAMA Network Open.*

eTable 6. Association of Health Information Exchange Status and Discharge Destination from Readmission, Medicare Beneficiaries without Alzheimer's Disease, 2018

Discharge Destination	Demographics & Baseline Clinical	Full
Home	0.93 (0.88, 0.97)	0.90 (0.86, 0.95)
Skilled Nursing Facility	1.03 (0.98, 1.08)	1.03 (0.98, 1.08)
Home w/ Home Health	1.11 (1.05, 1.17)	1.08 (1.02, 1.14)
Hospice	1.08 (0.99, 1.19)	1.04 (0.95, 1.14)
Against Medical Advice	0.93 (0.73, 1.19)	0.92 (0.72, 1.18)
Died	1.00 (0.91, 1.09)	0.96 (0.87, 1.05)
Other	0.81 (0.74, 0.89)	1.00 (0.91, 1.10)

Reference: fragmented admission-readmission pairs where the admission and readmission hospitals do not participate in the same HIE; each model includes hospital referral region random effects

Unadjusted

Model 1: demographics (age, sex, race), clinical (frailty score, Charlson comorbidity index, reason for readmission [DRG of readmission]), ***admitted from emergency department v other location***

Model 2: hospital (urban/rural, size, ownership, control, teaching; each for readmission hospital)

Model 3: Full model—all above covariates included

© 2023 Turbow SD et al. *JAMA Network Open.*

eTable 7. Association of Health Information Exchange Status and Discharge Destination from Readmission, Medicare Beneficiaries with Alzheimer's Disease, 2018

Discharge Destination	Demographics & Baseline Clinical	Full
Home	0.95 (0.84, 1.08)	0.92 (0.81, 1.05)
Skilled Nursing Facility	1.03 (0.91, 1.16)	1.02 (0.09, 1.15)
Home w/ Home Health	1.15 (1.01, 1.31)	1.14 (1.00, 1.30)
Hospice	1.08 (0.87, 1.35)	1.07 (0.86, 1.34)
Against Medical Advice	0.69 (0.33, 1.45)	0.69 (0.33, 1.47)
Died	0.82 (0.65, 1.04)	0.79 (0.62, 1.00)
Other	0.77 (0.61, 0.97)	0.96 (0.76, 1.21)

Reference: fragmented admission-readmission pairs where the admission and readmission hospitals do not participate in the same HIE; each model includes hospital referral region random effects
Unadjusted
Model 1: demographics (age, sex, race), clinical (frailty score, Charlson comorbidity index, reason for readmission [DRG of readmission]), ***admitted from emergency department v other location***
Model 2: hospital (urban/rural, size, ownership, control, teaching; each for readmission hospital)
Model 3: Full model—all above covariates included

eTable 8. Association of Health Information Exchange (HIE) Status and Discharge Destination From Readmission, Missing HIE Information Pairs Removed, Medicare Beneficiaries With and Without Alzheimer Disease, 2018

Discharge Destination	Full Model—patients without Alzheimer's Disease	Full Model—patients with Alzheimer's Disease
Home	0.93 (0.88, 0.98)	0.94 (0.82, 1.07)
Skilled Nursing Facility	1.00 (0.96, 1.08)	0.99 (0.89, 1.12)
Home w/ Home Health	1.10 (1.04, 1.16)	1.15 (1.01, 1.32)
Hospice	1.01 (0.92, 1.10)	1.05 (0.83, 1.31)
Against Medical Advice	0.88 (0.69, 1.13)	0.66 (0.31, 1.39)
Died	0.94 (0.85, 1.03)	0.77 (0.61, 0.98)
Other	1.02 (0.93, 1.11)	0.99 (0.79, 1.25)

Reference: fragmented admission-readmission pairs where the admission and readmission hospitals do not participate in the same HIE; each model includes hospital referral region random effects
Full Model: demographics (age, sex, race), clinical (frailty score, Charlson comorbidity index, reason for readmission [DRG of readmission]), hospital (urban/rural, size, ownership, control, teaching; each for readmission hospital)

© 2023 Turbow SD et al. *JAMA Network Open.*

eTable 9. Association of Health Information Exchange Status and Discharge Destination From Readmission Compared With Same Hospital/Nonfragmented Readmissions, Medicare Beneficiaries Without Alzheimer Disease, 2018

Discharge Destination		Unadjusted	Demographics & Baseline Clinical	Hospital	Full
Home	Same HIE*	1.02 (0.98, 1.07)	0.94 (0.90, 0.99)	1.00 (0.96, 1.05)	0.93 (0.89, 0.97)
	No Information Shared	1.03 (1.02, 1.05)	0.98 (0.96, 1.00)	1.04 (1.01, 1.06)	0.98 (0.96, 1.00)
Skilled Nursing Facility	Same HIE	1.02 (0.98, 1.06)	1.07 (1.02, 1.11)	1.04 (0.99, 1.08)	1.08 (1.04, 1.13)
	No Information Shared	1.03 (1.01, 1.05)	1.05 (1.05, 1.08)	1.04 (1.02, 1.06)	1.07 (1.05, 1.09)
Home Health	Same HIE	0.89 (0.85, 0.94)	0.90 (0.86, 0.94)	0.86 (0.82, 0.90)	0.86 (0.82, 0.91)
	No Information Shared	0.82 (0.80, 0.84)	0.82 (0.81, 0.84)	0.81 (0.79, 0.83)	0.82 (0.80, 0.84)
Hospice	Same HIE	0.89 (0.82, 0.97)	0.96 (0.88, 1.05)	0.90 (0.83, 0.98)	0.97 (0.89, 1.06)
	No Information Shared	0.88 (0.85, 0.92)	0.94 (0.90, 0.98)	0.91 (0.88, 0.95)	0.98 (0.94, 1.02)
Against Medical Advice	Same HIE	1.43 (1.14, 1.80)	1.31 (1.03, 1.66)	1.44 (1.15, 1.81)	1.33 (1.05, 1.68)
	No Information Shared	1.55 (1.39, 1.72)	1.48 (1.32, 1.65)	1.57 (1.40, 1.75)	1.48 (1.32, 1.66)
Died	Same HIE	1.21 (1.03, 1.22)	1.17 (1.07, 1.28)	1.12 (1.03, 1.22)	1.17 (1.07, 1.28)
	No Information Shared	1.13 (1.08, 1.17)	1.17 (1.12, 1.22)	1.15 (1.11, 1.20)	1.20 (1.15, 1.25)
Other	Same HIE	1.22 (1.12, 1.33)	1.21 (1.11, 1.31)	1.35 (1.24, 1.47)	1.33 (1.22, 1.46)
	No Information Shared	1.42 (1.36, 1.48)	1.40 (1.35, 1.46)	1.26 (1.21, 1.32)	1.25 (1.20, 1.30)

**HIE=health information exchange*

Reference: nonfragmented admission-readmission pairs; each model includes hospital referral region random effects

Unadjusted

Model 1: demographics (age, sex, race), clinical (frailty score, Charlson comorbidity index, reason for readmission [DRG of readmission])

Model 2: hospital (urban/rural, size, ownership, control, teaching; each for readmission hospital)

Model 3: Full model—all above covariates included

© 2023 Turbow SD et al. *JAMA Network Open.*

eTable 10. Association of Health Information Exchange Status and Discharge Destination From Readmission Compared With Same Hospital/Nonfragmented Readmissions, Medicare Beneficiaries With Alzheimer Disease, 2018

Discharge Destination		Unadjusted	Demographics & Baseline Clinical	Hospital	Full
Home	Same HIE*	1.09 (0.97, 1.23)	1.04 (0.92, 1.17)	1.08 (0.95, 1.21)	1.02 (0.91, 1.16)
	No Information Shared	1.09 (1.03, 1.15)	1.04 (0.98, 1.10)	1.11 (1.05, 1.17)	1.05 (0.99, 1.12)
Skilled Nursing Facility	Same HIE	0.98 (0.88, 1.10)	1.02 (0.91, 1.14)	0.99 (0.89, 1.11)	1.03 (0.92, 1.15)
	No Information Shared	0.99 (0.95, 1.05)	1.02 (0.96, 1.07)	1.00 (0.96, 1.06)	1.03 (0.98, 1.09)
Home Health	Same HIE	0.90 (0.80, 1.02)	0.90 (0.77, 1.02)	0.86 (0.76, 0.98)	0.87 (0.76, 0.98)
	No Information Shared	0.78 (0.74, 0.83)	0.79 (0.74, 0.83)	0.77 (0.72, 0.82)	0.77 (0.73, 0.82)
Hospice	Same HIE	0.90 (0.74, 1.11)	0.94 (0.76, 1.15)	0.91 (0.74, 1.11)	0.94 (0.76, 1.15)
	No Information Shared	0.84 (0.76, 0.93)	0.89 (0.80, 0.98)	0.86 (0.78, 0.95)	0.92 (0.83, 1.01)
Against Medical Advice	Same HIE	1.41 (0.71, 2.79)	1.16 (0.56, 2.41)	1.43 (0.72, 2.85)	1.19 (0.57, 2.48)
	No Information Shared	1.92 (1.43, 2.59)	1.77 (1.31, 2.40)	1.86 (1.37, 2.52)	1.70 (1.25, 2.32)
Died	Same HIE	0.92 (0.74, 1.15)	0.92 (0.73, 1.15)	0.92 (0.74, 1.16)	0.92 (0.73, 1.16)
	No Information Shared	1.10 (1.00, 1.21)	1.14 (1.03, 1.26)	1.13 (1.02, 1.25)	1.17 (1.06, 1.30)
Other	Same HIE	1.38 (1.11, 1.72)	1.37 (1.10, 1.71)	1.56 (1.25, 1.95)	1.56 (1.25, 1.95)
	No Information Shared	1.64 (1.48, 1.81)	1.60 (1.44, 1.77)	1.47 (1.33, 1.63)	1.44 (1.30, 1.60)

**HIE=health information exchange*

Reference: nonfragmented admission-readmission pairs; each model includes hospital referral region random effects

Unadjusted

Model 1: demographics (age, sex, race), clinical (frailty score, Charlson comorbidity index, reason for readmission [DRG of readmission])

Model 2: hospital (urban/rural, size, ownership, control, teaching; each for readmission hospital)

Model 3: Full model—all above covariates included

© 2023 Turbow SD et al. *JAMA Network Open.*

eTable 11. Association of Fragmented Readmissions and Discharge to Home Hospice or Facility-Based Hospice From Readmission, Medicare Beneficiaries, 2018

Alzheimer's Disease (AD) Status	Discharge Destination	Unadjusted	Demographics & Baseline Clinical	Hospital	Full
No AD	Home Hospice	0.85 (0.74, 0.97)	0.86 (0.75, 0.99)	0.84 (0.74, 0.96)	0.85 (0.74, 0.97)
	Facility Hospice	1.18 (1.05, 1.33)	1.21 (1.07, 1.36)	1.14 (1.01, 1.29)	1.15 (1.02, 1.30)
AD	Home Hospice	1.15 (0.86, 1.54)	1.11 (0.83, 1.49)	1.16 (0.86, 1.56)	1.11 (0.83, 1.50)
	Facility Hospice	1.01 (0.74, 1.38)	0.99 (0.72, 1.36)	1.00 (0.73, 1.38)	0.97 (0.70, 1.34)

Reference: fragmented admission-readmission pairs where the admission and readmission hospitals do not participate in the same HIE
Unadjusted
Model 1: demographics (age, sex, race), clinical (frailty score, Charlson comorbidity index, reason for readmission [DRG of readmission])
Model 2: hospital (urban/rural, size, ownership, control, teaching; each for readmission hospital)
Model 3: Full model—all above covariates included

© 2023 Turbow SD et al. *JAMA Network Open.*
