# Supplementary material for: Association of Fragmented Readmissions and Electronic Information Sharing With Discharge Destination Among Older Adults
Source: JAMA Netw Open. 2023 May 16;6(5):e2313592. doi: 10.1001/jamanetworkopen.2023.13592 (PMC10189568; doi:10.1001/jamanetworkopen.2023.13592)
Supplement: Supplement 2. — Data Sharing Statement [file jamanetwopen-e2313592-s002.pdf]

Data Sharing Statement

Turbow. Association of Fragmented Readmissions and Electronic Information Sharing With Discharge Destination Among Older Adults. *JAMA Netw Open*. Published May 16, 2023. doi:10.1001/jamanetworkopen.2023.13592

Data
Data available: No

Additional Information
Explanation for why data not available: This study used Medicare Research Identifiable Files, which were used under a data use agreement (DUA) with the Centers for Medicare and Medicaid Services. This DUA does not allow data sharing, so the data from this study will not be shared. The original data is available from the Centers for Medicare and Medicaid Services.
